# High-Efficiency and High-Quality Extraction of Hemicellulose of Bamboo by Freeze-Thaw Assisted Two-Step Alkali Treatment

**DOI:** 10.3390/ijms23158612

**Published:** 2022-08-03

**Authors:** Xin Wang, Jiahao He, Shuyu Pang, Shuangquan Yao, Chunxia Zhu, Jinwei Zhao, Yang Liu, Chen Liang, Chengrong Qin

**Affiliations:** Guangxi Key Laboratory of Clean Pulp & Papermaking and Pollution Control, School of Light Industrial and Food Engineering, Guangxi University, Nanning 530004, China; 2016301031@st.gxu.edu.cn (X.W.); 2016391011@st.gxu.edu.cn (J.H.); 2116301042@st.gxu.edu.cn (S.P.); yaoshuangquan@gxu.edu.cn (S.Y.); 2016301052@st.gxu.edu.cn (C.Z.); 1916391038@st.gxu.edu.cn (J.Z.); xiaobai@gxu.edu.cn (Y.L.); qinchengrong@gxu.edu.cn (C.Q.)

**Keywords:** high quality, extraction, hemicellulose, freeze–thaw, two-step alkali treatment

## Abstract

Hemicellulose is a major component of the complex biomass recalcitrance structure of fiber cell walls. Even though biomass recalcitrance protects plants, it affects the effective utilization of lignocellulosic biomass resources. Therefore, the separation and extraction of hemicellulose is very important. In this study, an improved two-step alkali pretreatment method was proposed to separate hemicellulose efficiently. Firstly, 16.61% hemicellulose was extracted from bamboo by the weak alkali treatment. Then, the physical freezing and the alkali treatment were carried out by freezing at −20 °C for 12.0 h and thawing at room temperature, heating to 80 °C, and treating with 5.0% sodium hydroxide for 90 min; the extraction yield of hemicellulose reached 73.93%. The total extraction yield of the two steps was 90.54%, and the molecular weight and purity reached 44,865 g·mol^–1^ and 89.60%, respectively. It provides a new method for breaking the biomass recalcitrance of wood fiber resources and effectively extracting hemicellulose.

## 1. Introduction

Lignocellulosic biomass is the most abundant natural renewable resource. It is a unique, low-cost sustainable resource that is widely used for the production of fuel, polymer materials, and chemicals [1]. Hemicellulose is an important component of lignocellulosic biomass [2]. Hemicellulose is hydrogen bonded with cellulose in plant cell walls and forms a lignin–carbohydrate complex (LCC) through a covalent bond with lignin [3]. LCC can protect wood fibers from biological invasion by forming biomass recalcitrance [4]. The “biomass recalcitrance” is derived from the complex three-dimensional structure formed during plant growth. However, the existence of biomass recalcitrance is not conducive to the concept utilization of full component biomass resources. Therefore, the extraction of hemicellulose from biomass resources is beneficial for breaking the biomass recalcitrance. The methods currently employed for extracting hemicellulose, such as hydrothermal treatments, acid treatments, alkali treatments, and organic solvent treatments [5], have certain disadvantages. Though the highest extraction yield is achieved with acid-treated hemicellulose, the further degradation of carbohydrates in hydrolysate causes the separation of hemicellulose to be difficult [6]. Moreover, the smaller molecular weight of extracted hemicellulose is not conducive to subsequent application. However, hemicellulose extraction using the hydrothermal method is advantageous in terms of the simplicity of the device employed and the usage of non-toxic or unharmful substances [7]. The hydrothermal method requires high temperature and pressure conditions, with the disadvantages of low extraction efficiency and a long, extended reaction time. Furthermore, the hydrothermal method is similar to the weak-acid treatment method [8], wherein a part of the hemicellulose degrades to monosaccharides owing to the increase in the acidity of the hydrolysate mixture. Hemicellulose also has a low degree of polymerization, which is not conducive to the subsequent extraction and purification. Despite the fact that high-purity hemicellulose can be obtained through organic solvent treatments, the common use of dimethyl sulfoxide and dioxane has demerits such as environmental pollution [9]. Saake et al. [10] reported that although the hemicellulose extracted using a 90.0% dimethyl sulfoxide solution has high purity, the extraction yield is low. However, most organic solvents are toxic and cause serious environmental pollution.

It has been demonstrated that hemicellulose can be obtained with a high yield and high molecular weight by alkali treatments at a low temperature and pressure [11]. Sun et al. [12] first ground wheat straw and then treated it with 4.0% KOH for 2.5 h at 35 °C and reported the extraction yield of hemicellulose and lignin to be 24.80% and 7.50%, respectively. Xu et al. [13] treated bagasse with 4.0% NaOH at 40 °C for 18.0 h and obtained hemicellulose with an extraction yield of 62.10%. However, conventional alkali treatment methods cause lignin to become highly soluble in alkali solutions, resulting in the separation and purification of hemicellulose becoming challenging. Further, the complex structure of lignocellulosic biomass causes difficulties in extracting and further utilizing hemicellulose. Therefore, many hemicellulose extraction processes that provide high-purity hemicellulose with high extraction yield have been developed. Peng et al. [14] successively extracted bamboo using distilled water, alkali, and organic alkali solvents (1.0% NaOH and 60.0% ethanol). At 60 °C, the samples extracted using organic alkali were treated with 1.0, 3.0, 5.0, and 8.0% NaOH for 3.0 h. The total extraction yield of hemicellulose reached 80.10%. Sun et al. [15] pretreated *Populus tomentosa* by steam explosion before and after soaking, following which it was subjected to alkali extraction and alkali/ethanol extraction. At 75 °C, *Populus tomentosa* was treated with 0.3, 0.6, 1.0, 1.5, and 2.5% KOH for 3.0 h, and the hemicellulose extraction yield was 76.30%. Jnawali et al. [16] extracted oligomeric hemicellulose from coconut shells using the alkali treatment combined with steam. When the coconut shells were treated with a 20.0% NaOH solution and steam for 1.0 h, 93.0% of the hemicellulose could be recovered. The aforementioned studies suggest that the extraction yield of hemicellulose can be improved by adopting a high alkali concentration or a multi-step treatment method. However, due to the influence of alkali concentration, the molecular weight of the extracted hemicellulose is relatively low. Therefore, while obtaining a high yield, it also has the characteristics of high molecular weight, which has become an important indicator for extracting hemicellulose. In the early stage of this research group [17], bamboo was frozen at −30 °C for 12.0 h, thawed at 25 °C, and then treated with 7.0% alkali at 75 °C for 90 min. The extraction yield of hemicellulose was 64.71%. Because physical freezing technology can provide a leaching channel for hemicellulose, the dissolution of hemicellulose is increased, but it needs further improvement. Therefore, we believe that the method of physical freezing combined with multi-step treatment can extract hemicellulose more effectively.

In this study, weak alkali was used for the preliminary treatment, then physical freezing technology was used to destroy the anti-degradation barrier of biomass, and finally, mild alkali conditions were used for the treatment. Through the improved two-step alkali treatment, the extraction yield of hemicellulose macromolecules was improved. The effects of the freezing conditions (temperature and time) and alkali treatment conditions (concentration, temperature, and time) on lignocellulose dissolution were studied. The physical and chemical structures before and after the treatment were analyzed using Fourier-transform infrared (FTIR), X-ray diffraction (XRD), and scanning electron microscopy (SEM) spectroscopy. The purity of the hemicellulose was analyzed by ion chromatography (IC), and the molecular weight was analyzed by gel permeation chromatography (GPC). This study provides a mild and efficient method to obtain high yield and high molecular weight hemicellulose while effectively breaking the biomass recalcitrance of fibrous cell walls.

## 2. Results and Discussion

### 2.1. Effects of Freezing Treatment and Alkaline Treatment on Hemicellulose Extraction

**Figure 1:**
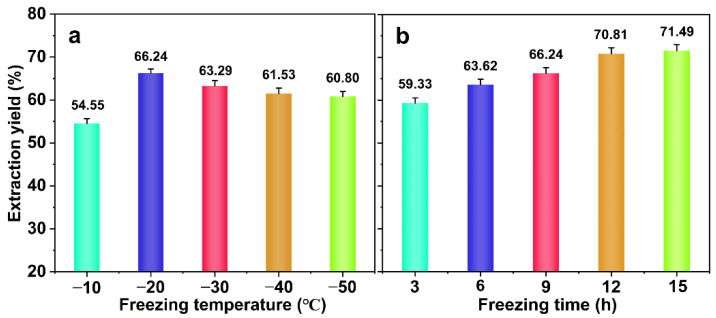
Effect of the freezing treatment on the extraction yield of hemicellulose (**a**, effect of freezing temperature; (**b**), effect of freezing time. The error bars represent the mean ± SD of samples over three replicates).

To the best of our knowledge, water expands by 9.0% below the freezing point. Accordingly, the volume of water increases when it is in a closed space [18]. The water pressure increases owing to the formation of ice crystals, and the cell walls of the bamboo rupture under this pressure [19]. Theoretically, this is conducive to the entry of chemicals into the bamboo. However, the growth of ice crystals was affected by different conditions [20]. The freezing conditions include temperature and time. Therefore, the effect of the freezing temperature and time on the extraction of hemicellulose was studied. The alkali treatment conditions are alkali concentration 5%, temperature 80 °C, time 75 min. See Figure 1 for details.

As shown in Figure 1a, with the decreasing freezing temperature, the extraction yield of hemicellulose increased first and then decreased. When the freezing temperature was −20 °C, the extraction yield of hemicellulose reached 66.24%. This result indicated that a suitable freezing temperature was needed for the extraction of hemicellulose. Ice crystals form inside objects in different numbers and sizes at different freezing temperatures; thus, the extent of the freezing-induced damage also varies [21]. The expansion of the ice crystal volume is affected by the freezing rate. The lower the temperature, the higher the freezing rate, and the larger the ice crystal volume. The formation of large ice crystals causes mechanical damage to the cell wall and thus enhances its alkali permeability [22]. However, the degradation and dissolution of lignin and cellulose in bamboo are intensified in this process. The migration channels for alkali liquor are provided by the fine and evenly distributed ice crystals, which facilitate alkali liquor infiltration [17].

With the extension of the freezing time, the extraction yield of hemicellulose showed a trend to gradually increase. See Figure 1b. However, when the freezing time was greater than 12.0 h (extraction yield, 70.81%), the extraction yield increased more slowly. This is due to the increase in the size and number of ice crystals growing inside the bamboo with the increase in the freezing time. Between 3.0 h and 12.0 h, as the freezing time increases, the formation of fine ice crystals was promoted [18]. When freezing for 12.0 h, the quantity of the channels through which the alkali liquor can penetrate was increased because the fine ice crystals were evenly distributed inside the bamboo. Consequently, hemicellulose was extracted in a large amount. When frozen for more than 12.0 h, the small ice crystals grew into larger ones [23]. The physical damage to the cell wall by the ice crystals only increased locally, resulting in the change in hemicellulose extraction was not obvious. The results showed that freezing conditions at −20 °C and 12.0 h were more favorable for the extraction of hemicellulose.

**Figure 2:**
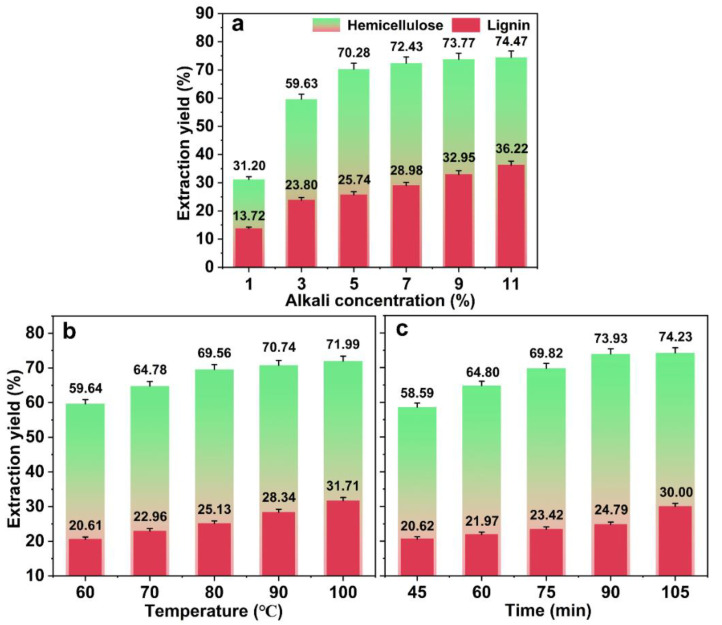
Effect of the alkali treatment on the extraction yield of hemicellulose and lignin ((**a**), effect of alkali concentration; (**b**), effect of temperature. (**c**), effect of time. The error bars represent the mean ± SD of samples over three replicates).

After the freezing conditions (temperature −20 °C, time 12 h) were determined, the alkali concentration, temperature, and time were studied. See Figure 2 for details.

The effect of the alkali concentration was explored under the condition of an alkali treatment temperature of 80 °C and time of 80 min, as shown in Figure 2a. The extraction yield of hemicellulose changed obviously with the alkali concentration, which increased from 31.20% at 1.0% alkali concentration to 70.28% at 5.0% alkali concentration. When the alkali concentration was more than 5.0%, the extraction yield of hemicellulose did not increase significantly and reached 74.47% at an 11.0% alkali concentration. This result indicated that the extraction of hemicellulose was limited at high alkali concentrations. After the freezing pretreatment, the number of pores on the bamboo surface increased, and the infiltration effect of the alkali liquor was enhanced [22]. Consequently, the strength of the action of the alkali liquor on hemicellulose was increased. Although the extraction yield of hemicellulose increased with the increasing alkali liquor concentration, the hemicellulose exposed to the alkali solution was gradually consumed. When the alkali concentration continued to increase, the dissolution amount of hemicellulose did not change significantly, and the extraction yield remained stable. The extraction yield of lignin increased from 13.72% to 36.22%, with an increase in the alkali liquor concentration. This was due to the dissolution of lignin in the alkaline solution [24]. However, when the alkali concentration exceeded 5.0%, the dissolution of lignin led to the difficult separation of hemicellulose in the hydrolysate. The utilization of high-value residual solids is affected.

The effect of temperature was explored under the condition of an alkali treatment concentration of 5% and a time of 80 min. The extraction yield of hemicellulose increased from 59.64% to 71.99%, with an increase in temperature from 60 °C to 80 °C (See Figure 2b). However, the extraction yield of hemicellulose did not increase significantly at temperatures above 80 °C. This is due to the extensive removal of hemicellulose exposed to the alkali solution at 80 °C. As the temperature was increased further, the extraction yield of hemicellulose increased only slightly. The dissolution of lignin also increased with increasing temperature. This result indicated that a high temperature was more suitable for lignin extraction [25].

The effect of time was investigated under the condition of an alkali treatment concentration of 5% and a temperature of 80 °C. The extraction yield of hemicellulose increased from 58.59% to 75.83%, with an increase in the treatment time from 45 min to 105 min, see Figure 2c. This is attributed to the rapid dissolution of free hemicellulose by the permeation of the alkali liquor. At the same time, the hemicellulose, which is hydrogen-bonded with cellulose, cannot be dissolved sufficiently [26]. Therefore, the dissolution of hemicellulose was promoted with prolonged alkali treatment time. When the alkali treatment time exceeded 90 min, the dissolution change of hemicellulose was not obvious. This is due to the gradual dissolution of hemicellulose with a complex structure. The dissolution of hemicellulose decreased upon prolonging the alkali treatment time. The extraction yield of lignin also increased with the increasing alkali treatment time; it increased from 20.62% to 30.0%. Anti-degradation shield breakage and alkali penetration are promoted when the pretreatment time is prolonged [27]. This causes more lignin to be exposed to the alkali liquor. Therefore, the lignin dissolution rate increased with time.

According to the results, the optimal freeze–thaw-assisted alkali treatment conditions are as follows: a freezing condition of (temperature, −20 °C; time, 12.0 h) and an alkali treatment condition of (concentration, 5.0%; temperature, 80 °C; time, 90 min), respectively. Under these conditions, the total extraction yield of hemicellulose and lignin were 90.54% and 24.79%, respectively. This method is a milder and more efficient extraction method for hemicellulose compared with other hemicellulose extraction methods (refer to Table 1 for details) [14,15,16,17].

### 2.2. Characterization of the Residual Solids

Hemicellulose was effectively extracted by the weak-alkali pre-treatment, followed by freeze–thaw alkali extraction. The other components were less affected. Therefore, the structural analysis of the treated bamboo residue was carried out. The examined characteristics mainly included surface morphology, functional groups, and crystallinity.

**Figure 3:**
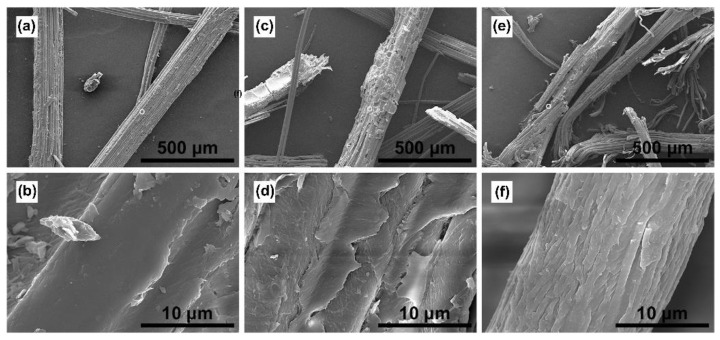
SEM of bamboo before and after different treatments ((**a**,**b**), Raw material; (**c**,**d**), after weak-alkali treatment; and (**e**,**f**), freeze–thaw-assisted alkali treatment).

The apparent morphologies of the bamboo powder before the treatment, after the weak-alkali treatment, and after the weak-alkali/freeze–thaw-assisted alkali treatment were characterized using SEM (see Figure 3).

The fibers on the surface of the raw bamboo material were closely arranged and covered with pectin (See Figure 3a,b). After the weak-alkali treatment, the fiber arrangement became relatively compact, and the surface of the bamboo powder turned rough (See Figure 3c,d). A small number of minor cracks appeared on the surface of the sample, and the covering material was partially destroyed. The alkali solution could penetrate the fiber surface and destroy the anti-degradation barrier to a certain extent and thus assist the subsequent dissolution of hemicellulose. After the freeze–thaw pretreatment and alkali treatment, the fiber bundles in the bamboo became loose (See Figure 3e,f). The anti-degradation barrier was destroyed, and the fiber bundles were looser and exposed. The extraction of hemicellulose using this method is efficient and has little effect on the dissolution of lignin and cellulose. Therefore, the fiber integrity was preserved.

**Figure 4:**
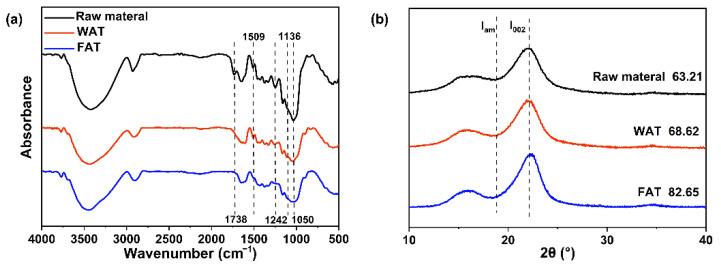
Changes of main functional groups and crystallinity index of bamboo with and without different treatment (**a**), Fourier transform infrared (FTIR) spectra; (**b**), X-ray diffraction (XRD) patterns; WAT, Weak alkali treatment; FAT, Freeze–thaw assisted alkali treatment).

The changes in the main functional groups of the raw material and the solids remaining after the treatments were analyzed using FTIR (see Figure 4a). The vibrations of the carbonyl and acetyl groups of hemicellulose lead to an absorption peak at 1738 cm^−1^ [28]. After treatment, hemicellulose was largely dissolved; therefore, its absorption peaks did not appear in the spectrum of the residual solid. The stretching vibration of the aromatic ring of lignin results in an absorption peak at 1509 cm^−1^. The absorption peak intensity did not change significantly after pretreatment compared with that of the raw material. The vibration of the ether bond in lignin produces an absorption peak at 1242 cm^−1^ [29]. After the weak-alkali treatment and the freeze–thaw alkali extraction, the intensity of the absorption peaks decreased gradually. This means that the ether bond of lignin was broken, and part of the lignin was dissolved. The C-H stretching vibration of aromatic nuclei has an absorption peak at 1136 cm^−1^. The absorption peak intensity of xylan decreased significantly after the alkali treatment because of the dissolution of hemicellulose during the treatment. The C-O vibration of hemicellulose causes the absorption peak at 1050 cm^−1^ [30]. The raw material has an obvious absorption peak here. The absorption peak weakened after the weak-alkali treatment. After the freeze–thaw alkali treatment, the signals disappeared owing to the removal of a large amount of hemicellulose. The spectral results are consistent with other experimental results and indicate that the hemicellulose extraction was effective [31].

Under the conditions of the alkali treatment, the main components of bamboo were dissolved in sequence. The crystallinity of the cellulose in the sample varies with the processing conditions. For the XRD patterns of the remaining solids after the weak-alkali treatment and the subsequent freeze–thaw alkali extraction see Figure 4b. The three curves having the same shape represent the typical diffraction peaks of cellulose I, but the peak intensities are different. The maximum peak strength of the I_002_ peak for the untreated sample is 1098, while the maximum I_002_ peak strengths for the residual solids obtained after weak-alkali pretreatment and freeze–thaw-assisted alkali treatment are 1211 and 1349, respectively. The peak intensities of the amorphous region I_am_ are 404, 380, and 234. The alkali treatment had obvious changes in the aggregation of the amorphous components of bamboo compared with that in the raw material. The Crl of the material before the treatment, after the weak-alkali pretreatment, and after the freeze–thaw alkali treatment were determined to be 63.21%, 68.62%, and 82.65%, respectively. This is because of the effective removal of a number of hemicellulose from the amorphous region by the freeze–thaw alkali treatment. Compared to hemicellulose, the other major components were less soluble. Therefore, the relative cellulose content of the remaining solids increased.

### 2.3. Characterization of the Hemicellulose

The criteria for judging the quality of hemicellulose were mainly reflected in two factors: molecular weight and purity.

The molecular weights of the hemicellulose obtained after the different treatments were analyzed by GPC. The weight-average molecular weights (*M*w) of hemicellulose after the weak alkali pretreatment and freeze–thaw-assisted alkali treatment were 38,552 and 44,865 g·mol^–1^, respectively. This indicated that the low-molecular-weight hemicellulose was extracted during the one-step weak-alkali pretreatment, but the extraction yield was only 16.61%. The molecular weight of hemicellulose after the freeze–thaw alkali treatment was higher than that after the weak-alkali pretreatment. Ice crystals destroy the bonds between hemicellulose and other components more thoroughly, which is beneficial to the extraction of macromolecular hemicellulose. The hemicellulose polydispersity index (*M*w/*M*n) after the freeze–thaw alkali treatment was low, at only 1.38. This indicated that the relative compositional uniformity of hemicellulose after the freeze–thaw alkali treatment was better. The molecular weight of hemicellulose (39,614 g·mol^–1^) and the extraction yield (64.71%) are better than those obtained using a previously-reported optimal extraction process [17].

The chemical composition of hemicellulose after different extraction treatments is analyzed by IC, and the results are presented in Table 2. The purity of the hemicellulose extracted by the weak-alkali treatment was 77.94%. The contents of xylose, glucose, arabinose, and galactose were 47.01, 37.52, 11.48, and 3.99%, respectively. The high glucose content may be related to the presence of starch and α-glucan in the bamboo cell wall. Similar results have been reported previously [32]. The purity of hemicellulose extracted by the freeze–thaw alkali treatment was 89.60%. The contents of xylose, glucose, arabinose, and galactose were 60.18, 33.97, 4.39, and 1.46%, respectively. The sugars in the hemicellulose branch chain were partially degraded after the freeze–thaw alkali treatment, which is more conducive to xylose fermentation. The results showed that the obtained hemicellulose sample has good application prospects.

## 3. Materials and Methods

### 3.1. Materials and Reagents

The bamboos used in this study were procured from a local company (Guangxi, China). First, they were cut into small strips, which were dried naturally. Subsequently, the dried bamboos were ground in a grinder (ZVK-M20, Schmersal, Idar-Oberstein, Germany) to obtain a powder (40–60 mesh), which was stored in airtight bags until the next steps were performed.

Fifty percent sodium hydroxide solution was supplied from Thermo Fisher Scientific (Waltham, MA, USA). Other chemicals were purchased from Sigma-Aldrich (Milwaukee, WI, USA) and were of analytical grade.

### 3.2. Chemical Composition Analysis of the Bamboos

The main chemical composition of the bamboo powder (refer to Table 3) was analyzed according to the standardized method of the NREL (National Renewable Energy Laboratory) [33]. First, 300 mg of the 40–60 mesh bamboo powder was placed in a constant-temperature shaker (UF-92AM23, ZHICHU, Shanghai, China) at 30 °C and hydrolyzed with 72.0% H_2_SO_4_ for 1.0 h. Thereafter, the resulting mixture was diluted using 4.0% H_2_SO_4_ and heated in an autoclave (SX-500, TOMY, Tokyo, Japan) at 121 °C for 1.0 h. The obtained solid was hydrolyzed and filtered through a Buchner funnel. The remaining solid was naturally air-dried and weighed. The filtrate was used for the detection of sugar composition and acid-soluble lignin content, and the remaining solid was used for acid-insoluble lignin content detection. The final data are the average of the three identical experiments. The data were presented as values and are given as mean ± SD. 

### 3.3. Weak-Alkali Treatment

The bamboo powder was treated with a weak alkali solution in one step. The bamboo powder (20 g) and 0.5% (g/w) alkali solution (solid: liquid ratio, 1:8) were placed in a constant temperature reactor (VRD-42SD-A, CNPPRI, Beijing, China) at 90 °C for 60 min. The extracted liquid was used to further extract hemicellulose, and the residue was dried and weighed for further treatment. The extraction yield of hemicellulose was 16.61%. The extraction yield of lignin was 4.96%. The final data are the average of three identical experiments. The formula for calculating the extraction yield of hemicellulose and lignin is as follows:

H = (C_1_ − C_2_)/C_1_ × 100%

L = (C_3_ − C_4_)/C_3_ × 100%

In the formula, H is the hemicellulose yield %, C_1_ is the hemicellulose content % in the raw material, and C_2_ is the hemicellulose content % in the remaining solids. L is the lignin yield %, C_3_ is the lignin content % in the raw material, and C_4_ is the lignin content % in the remaining solids.

### 3.4. Freeze–Thaw Alkali Treatment of the Residual of Bamboo Powder

The freeze–thaw operations were conducted as follows: the bamboo powder residue obtained after the weak-alkali treatment was soaked in pure water (solid: liquid ratio, 1:8) at 25 °C for 12.0 h. Thereafter, the alkali solutions of different concentrations were added to the fully soaked samples. The samples were frozen at different temperatures (−10, −20, −30, −40, and −50 °C) for different times (3.0, 6.0, 9.0, 12.0, and 15.0 h) in an ultra-low temperature refrigerator (Eppendorf CryocubeF440, Hamburg, Germany) and subsequently defrosted at 25 °C for 12.0 h. The thawed bamboo powder samples were loaded into a constant temperature reactor and subjected to alkali treatment in a rotating digester thereafter (BS500, LAIBEI, Shanghai, China). The reactions were conducted at varying alkali concentrations (1.0, 3.0, 5.0, 7.0, 9.0, and 11.0%), temperatures (60, 70, 80, 90, and 100 °C), and time (45, 60, 75, 90, and 105 min) intervals. Finally, it enters the Buchner funnel for solid–liquid separation. The final data are the average of three identical experiments.

### 3.5. Characterization of the Structures of Solid Residue

The surface morphologies of the solid residue were analyzed by SEM (SU8020, Japanese Hi-Tech, Tokyo, Japan) before and after different treatments. The specific operation is as follows, first, apply the conductive adhesive on the sample stage. Then the bamboo powder sample is evenly sprinkled on the conductive glue. Afterwards, the unadhered samples were blown off with an ear-washing ball and treated with gold spraying. The changes in the main functional groups of the bamboo fiber treated under different conditions were analyzed by FTIR spectroscopy (IRTracer-100, Shimadzu Corporation, Tsushima, Japan) [34]. The crystallinity of the bamboo fibers was analyzed by XRD (Rigaku D/MAX 2500V, Rigaku Corporation, Tokyo, Japan) [33].

### 3.6. Detection of Lignin Content

The samples were subjected to two-step acid hydrolysis. After the acid hydrolyzate was cooled to room temperature, the solid acid-insoluble lignin and the acid-soluble lignin in the liquid were separated by filtration using a G3 glass filter that was dried at 105 °C to constant weight in advance. The acid-insoluble lignin mass was obtained from the weight difference in the G3 glass filter. Acid-soluble lignin was detected by UV-Vis (Agilent 8453, Agilent, Palo Alto, CA, USA). The final data are the average of three identical experiments. 

### 3.7. Analysis of Hemicellulose

The separated extract was adjusted to pH 2 with 1.0% hydrochloric acid. Precipitate with 3 volumes of ethanol and freeze-dried for testing. The specific operation was 50 mg of hemicellulose was each accurately weighed and dissolved in 3 mL of 72% sulfuric acid. Then it was oscillated for 1 h at 30 °C and 150 rpm. The sulfuric acid concentration was diluted to 4% after one-step acid hydrolysis. Finally, the reaction was performed at 121 °C for 1 h in a high-temperature autoclave. The acid-hydrolyzed liquid was taken and centrifuged for a sugar composition test. The purity of the extracted hemicellulose was analyzed by IC (ICS-5000 Plus, Diane, Albuquerque, USA) using a Dionex carbopac PA20 chromatographic column (3 mm × 150 mm). The final data are the average of three identical experiments. The data were presented as values and are given as mean ± SD. The formula for calculating the content of sugar components in the sample is as follows:

A = (C × 0.261 × N)/0.05

where A is the monosaccharide content in the sample %; C is monosaccharide concentration, g/L; N is the dilution multiple; 0.261 is the solution volume L during acidolysis; 50 is the absolute dry mass of acidolysis sample, g.

The weight-average molecular weight (*M*_W_) and number-average molecular weight (Mn) distributions of each hemicellulose sample were determined by gel permeation chromatography (GPC). Then, 10 mg of the sample was weighed and dissolved in 10 mL of sodium phosphate-buffered saline, shaken well, and left to stand. Then, 100 μL of the prepared solution was injected into Agilent PL-GPC220 (Agilent, Palo Alto, CA, USA) for detection and analysis.

### 3.8. Statistical Analysis

The statistical calculations were performed using Microsoft Excel and IBM SPSS Statistics 19 (IBM, Armonk, NY, USA). A one-way analysis of variance (ANOVA) was used to compare the treatment means. The data were presented as mean ± SD. Statistical analysis of variance was calculated using a Student’s t-test. Triplicate experiments were performed for all sample tests.

## 4. Conclusions

The weak-alkali pretreatment combined with freeze–thaw-assisted alkali treatment is a new, mild, and efficient pretreatment method for extracting hemicellulose. The extraction yield of hemicellulose reached 90.54% under the following conditions: freezing temperature and time were −20 °C and 12.0 h, alkali concentration of 5.0%, alkali treatment temperature and time were 80 °C and 90 min. Various characterization results indicated that hemicellulose was extracted in large quantities under the optimized conditions. Using this method, highly-pure hemicellulose samples of high molecular weights can be obtained in high yields. It provides a theoretical basis for breaking the biomass recalcitrance of fiber cell walls.

## Figures and Tables

**Table 1:** Extraction yield of hemicellulose after different treatments.

Raw Material	Extraction Method	Extraction Yield (%)
Poplar [15]	Steam explosion, alkali and alkali/ethanol were treated at 75 °C for 3.0 h with 0.3%, 0.6%, 1.0%,1.5% and 2.5% KOH.	76.40
Coconut shell [16]	Combined treatment with 20% NaOH and steam for 1.0 h.	93.0
Bamboo [14]	Distilled water, alkali and organic solvents were treated with 1.0%, 3.0%, 5.0% and 8.0% NaOH for 3.0 h at 60 °C.	80.10
Bamboo [17]	−30 °C for 12.0 h, 7.0% NaOH treatment for 1.5 h at 75 °C.	64.71
Bamboo	Weak alkali treatment, −20 °C for 12.0 h, 5.0% NaOH treatment for 1.5 h at 80 °C.	90.54

**Table 2:** Extraction yield and purity of hemicellulose obtained by different treatments.

Samples	Extraction (%)	Purity (%)	Xylose (%)	Glucose (%)	Arabinose (%)	Galactose (%)
WAT	16.61 ± 0.66	77.94 ± 3.12	47.01 ± 1.88	37.52 ± 1.50	11.48 ± 0.46	3.99 ± 0.16
FAT	73.93 ± 2.96	89.60 ± 3.58	60.18 ± 2.41	33.97 ± 1.36	4.39 ± 0.17	1.46 ± 0.06

**Table 3:** Chemical composition of bamboo after different treatments.

Samples	Cellulose (%)	Hemicellulose (%)	Lignin (%)
Raw material	49.50 ± 2.47	17.19 ± 0.86	25.25 ± 1.26
WAT	47.75 ± 2.12 **	14.33 ± 0.72 **	24.00 ± 1.15 **
FAT	43.41 ± 2.03 **	4.47 ± 0.16 **	18.99 ± 0.89 **

** Statistically significant differences between the raw material and remaining solids after the treatment are marked with an asterisk (** *p* < 0.01; ANOVA).

## Data Availability

Not applicable.

## References

[1] Takkellapati S., Li T., Gonzalez M.A. (2018). An overview of biorefinery-derived platform chemicals from a cellulose and hemicellulose biorefinery. Clean Technol. Environ. Policy.

[2] Limayem A., Ricke S.C. (2012). Lignocellulosic biomass for bioethanol production: Current perspectives, potential issues and future prospects. Prog. Energy Combust. Sci..

[3] Giummarella N., Pu Y.Q., Ragauskas A.J., Lawoko M. (2019). A critical review on the analysis of lignin carbohydrate bonds. Green Chem..

[4] Dev A., Srivastava A.K., Karmakar S. (2018). Nanomaterial toxicity for plants. Environ. Chem. Lett..

[5] Kumar A.K., Sharma S. (2017). Recent updates on different methods of pretreatment of lignocellulosic feedstocks: A review. Bioresour. Bioprocess..

[6] Wang X.H., Zhang C.H., Lin Q.X., Cheng B.G., Kong F.G., Li H.L., Ren J.L. (2018). Solid acid-induced hydrothermal treatment of bagasse for production of furfural and levulinic acid by a two-step process. Ind. Crop. Prod..

[7] Yao S.Q., Nie S.X., Yuan Y., Wang S.F., Qin C.R. (2015). Efficient extraction of bagasse hemicelluloses and characterization of solid remainder. Bioresour. Technol..

[8] Methacanon P., Chaikumpollert O., Thavorniti P., Suchiva K. (2003). Hemicellulosic polymer from Vetiver grass and its physicochemical properties. Carbohydr. Polym..

[9] Maurya D.P., Singla A., Negi S. (2015). An overview of key pretreatment processes for biological conversion of lignocellulosic biomass to bioethanol. 3 Biotech.

[10] Saake B., Kruse T., Puls J. (2001). Investigation on molar mass, solubility and enzymatic fragmentation of xylans by multi-detected SEC chromatography. Bioresour. Technol..

[11] Svard A., Brannvall E., Edlund U. (2017). Rapeseed straw polymeric hemicelluloses obtained by extraction methods based on severity factor. Ind. Crop. Prod..

[12] Sun R.C., Tomkinson J., Ma P.L., Liang S.F. (2000). Comparative study of hemicelluloses from rice straw by alkali and hydrogen peroxide treatments. Carbohydr. Polym..

[13] Xu F., Sun J.X., Liu C.F., Sun R.C. (2006). Comparative study of alkali- and acidic organic solvent-soluble hemicellulosic polysaccharides from sugarcane bagasse. Carbohydr. Res..

[14] Peng P., Peng F., Bian J., Xu F., Sun R.C., Kennedy J.F. (2011). Isolation and structural characterization of hemicelluloses from the bamboo species Phyllostachys incarnata Wen. Carbohydr. Polym..

[15] Sun S.L., Wen J.L., Ma M.G., Sun R.C. (2013). Successive alkali extraction and structural characterization of hemicelluloses from sweet sorghum stem. Carbohydr. Polym..

[16] Jnawali P., Kumar V., Tanwar B., Hirdyani H., Gupta P. (2018). Enzymatic Production of Xylooligosaccharides from Brown Coconut Husk Treated with Sodium Hydroxide. Waste Biomass Valorization.

[17] Li J., Liu Z.M., Feng C.Q., Liu X.Y., Qin F.Y., Liang C., Bian H.Y., Qin C.R., Yao S.Q. (2021). Green, efficient extraction of bamboo hemicellulose using freeze-thaw assisted alkali treatment. Bioresour. Technol..

[18] Zhu H.X., Ma Q.L., Sheng J., Yang R.D. (2020). Freeze-thaw repetition as an auxiliary method to promote efficient separation of hemicellulose from poplar. Green Chem..

[19] Hallet B. (2006). Geology. Why do freezing rocks break?. Science.

[20] Erlandsson J., Pettersson T., Ingverud T., Granberg H., Larsson P.A., Malkoch M., Wagberg L. (2018). On the mechanism behind freezing-induced chemical crosslinking in ice-templated cellulose nanofibril aerogels. J. Mater. Chem. A.

[21] Zhang T.X., Wang L.L., Wang Z.J., Li J.J., Wang J.C. (2021). Single Ice Crystal Growth with Controlled Orientation during Directional Freezing. J. Phys. Chem. B.

[22] Ando Y., Hagiwara S., Nabetani H., Okunishi T., Okadome H. (2019). Impact of ice crystal development on electrical impedance characteristics and mechanical property of green asparagus stems. J. Food Eng..

[23] Gong Y.Y., Xu S.Y., He T., Dong R., Ren T., Wang X.L., Hu X.Z. (2020). Effect of quick-freezing temperature on starch retrogradation and ice crystals properties of steamed oat roll. J. Cereal Sci..

[24] Lappalainen J., Baudouin D., Hornung U., Schuler J., Melin K., Bjelic S., Vogel F., Konttinen J., Joronen T. (2020). Sub- and Supercritical Water Liquefaction of Kraft Lignin and Black Liquor Derived Lignin. Energies.

[25] El Hage R., Chrusciel L., Desharnais L., Brosse N. (2010). Effect of autohydrolysis of Miscanthus x giganteus on lignin structure and organosolv delignification. Bioresour. Technol..

[26] Li J.G., Hu H.C., Li H.L., Huang L.L., Chen L.H., Ni Y.H. (2017). Kinetics and mechanism of hemicelluloses removal from cellulosic fibers during the cold caustic extraction process. Bioresour. Technol..

[27] Brandt A., Ray M.J., To T.Q., Leak D.J., Murphy R.J., Welton T. (2011). Ionic liquid pretreatment of lignocellulosic biomass with ionic liquid-water mixtures. Green Chem..

[28] Sun X.F., Xu F., Zhao H., Sun R.C., Fowler P., Baird M.S. (2005). Physicochemical characterisation of residual hemicelluloses isolated with cyanamide-activated hydrogen peroxide from organosolv pre-treated wheat straw. Bioresour. Technol..

[29] Liu X., Wei W.Q., Wu S.B. (2019). Synergism of organic acid and deep eutectic solvents pretreatment for the co-production of oligosaccharides and enhancing enzymatic saccharification. Bioresour. Technol..

[30] Liu C.F., Sun R.C., Zhang A.P., Ren J.L. (2007). Preparation of sugarcane bagasse cellulosic phthalate using an ionic liquid as reaction medium. Carbohydr. Polym..

[31] Luo Y.D., Li Y., Cao L.M., Zhu J.T., Deng B.J., Hou Y.J., Liang C., Huang C.X., Qin C.R., Yao S.Q. (2021). High efficiency and clean separation of eucalyptus components by glycolic acid pretreatment. Bioresour. Technol..

[32] Peng P., Peng F., Bian J., Xu F., Sun R.C. (2011). Studies on the Starch and Hemicelluloses Fractionated by Graded Ethanol Precipitation from Bamboo Phyllostachys bambusoides f. shouzhu Yi. J. Agric. Food Chem..

[33] Ge J.Y., Wu Y.T., Han Y.S., Qin C.R., Nie S.X., Liu S.J., Wang S.F., Yao S.Q. (2020). Effect of hydrothermal pretreatment on the demineralization and thermal degradation behavior of eucalyptus. Bioresour. Technol..

[34] Jin X.C., Hu Z.H., Wu S.F., Song T., Yue F.X., Xiang Z.Y. (2019). Promoting the material properties of xylan-type hemicelluloses from the extraction step. Carbohydr. Polym..

